# Risk of drug use during pregnancy: master protocol for living systematic reviews and meta-analyses performed in the metaPreg project

**DOI:** 10.1186/s13643-023-02256-8

**Published:** 2023-06-21

**Authors:** Cyndie Picot, Priscilla Ajiji, Lucie Jurek, Mikail Nourredine, Jérôme Massardier, Audrey Peron, Michel Cucherat, Judith Cottin

**Affiliations:** 1 Service Hospitalo-Universitaire de Pharmaco-Toxicologie, Hospices Civils de Lyon, Bât. A-162, avenue Lacassagne, 69424 Cedex 03 Lyon, France; 2 Faculté de Santé, Université Paris-Est Créteil, EA 7379, Créteil, France; 3 French National Agency for Medicines and Health Products Safety (ANSM), Saint Denis, France; 4 Child and Adolescent Neurodevelopmental Psychiatry Department, Center for Assessment and Diagnostic of Autism, Le Vinatier Hospital, Bron, France; 5 RESHAPE, Université Claude Bernard Lyon 1, U1290 Lyon, France; 6 Service de biostatistiques, Hospices Civils de Lyon, Lyon, France; 7 Laboratoire d’évaluation et modélisation des effets thérapeutiques, UMR CNRS 5558, Lyon, France; 8 Service de Gynécologie Obstétrique et Médecine Foetale, HFME, Hospices Civils de Lyon, Lyon, France; 9 metaEvidence.org - Service Hospitalo, Universitaire de Pharmaco-Toxicologie, Hospices Civils de Lyon, Lyon, France

**Keywords:** Evidence synthesis, Living meta-analysis, Systematic review, Pregnancy, Drug safety, In utero drug exposure, Congenital malformations, Adverse birth outcome, Stillbirth, Birth defects

## Abstract

**Background:**

Knowledge about the risks of drugs during pregnancy is continuously evolving due to the frequent publication of a large number of epidemiological studies. Systematic reviews and meta-analyses therefore need to be regularly updated to reflect these advances. To improve dissemination of this updated information, we developed an initiative of real-time full-scale living meta-analyses relying on an open online dissemination platform (www.metapreg.org).

**Method:**

All living meta-analyses performed in this project will be conducted in accordance with this master protocol after adaptation of the search strategy. A systematic literature search of PubMed and Embase will be performed. All analytical studies (e.g., cohort, case-control, randomized studies) reporting original empirical findings on the association between in utero exposure to drugs and adverse pregnancy outcomes will be included. Study screening and data extraction will be performed in a semi-automation way supervised by a biocurator. A risk of bias will be assessed using the ROBINS-I tools. All clinically relevant pregnancy adverse outcomes (malformations, stillbirths, neuro-developmental disorders, pre-eclampsia, etc.) available in the included studies will be pooled through random-effects meta-analysis. Heterogeneity will be evaluated by *I*^2^ statistics.

**Discussion:**

Our living systematic reviews and subsequent updates will inform the medical, regulatory, and health policy communities as the news results evolve to guide decisions on the proper use of drugs during the pregnancy.

**Systematic review registration:**

Open Science Framework (OSF) registries

**Supplementary Information:**

The online version contains supplementary material available at 10.1186/s13643-023-02256-8.

## Background

In the last decades, the interest in the risk of drug use during pregnancy has increased, leading to an important research activity and a significant expansion in the number of epidemiological studies, mainly observational, available in the scientific literature. These studies are of paramount importance for both healthcare professionals, health authorities, and more generally in terms of public health. Take into account the results of the continuously published epidemiological studies, as soon as possible is imperative, especially when they provide evidence of relevant harm. The consequences of a wrong evaluation, based on out-of-date data, are so profound: falsely concluding to a risk that can result in women forgoing needed therapies, in unnecessary invasive diagnostics, and, in some cases, terminating wanted pregnancies; and failing to detect true risks of medicine exposure that can cause serious effects on the unborn child and have negative consequences in the population.

Therefore, although time-consuming, an extensive bibliographic search is imperative to detect newly published studies in a timely manner. However, two core elements impede decision-making for agencies, policy makers, and health professionals using these results to make real-time decisions:

- The infodemia that leads to having too much information including false or misleading information [1]. Indeed, as in all fields, the methodological quality of these studies is often debatable [2]. Therefore, selecting appropriate studies is essential for the decision-making and requires advanced expertise given the field of study and the observational nature of the available studies [3–7].
- The study results are sometimes conflicting between studies qualitatively (in terms of statistical significance) or quantitatively due to random sampling fluctuation or due to bias.

These issues are usually dealt with through systematic reviews and meta-analysis, which requires a quantitative synthesis to account for variability induced by random sampling fluctuations and heterogeneity in statistical power [8]. However, this approach also has its own limitations:

- Multiple systematic reviews and meta-analyses are published on the same topic. These ones are of varying quality and methodology (in the choice of eligible studies, selection of comparisons, populations, outcomes of interest, …) leading to contradictory results, contributing as their turn in an infodemia [9–11].
- Published meta-analyses quickly become obsolete because of (i) the continuous publication of new studies, (ii) the static nature of the journal publications, and (iii) the delay in publication. Indeed, it was estimated that the median publication time was about 15–16 months, from registration of a protocol to publication [12–14].
- Systematic reviews and meta-analyses are not systematically performed, and some clinical questions remain without synthesis for many years because no author addresses the subject. Indeed, it is usual to wait for the availability of several published studies before undertaking an evidence synthesis.

This situation demands that decision-makers and users of this information maintain a double scientific intelligence, of the studies themselves and of the syntheses [15–18]. The approach of living systematic review proposed by the Living Systematic Review Network [19] was developed in this context and was particularly important when research evidence is emerging rapidly, current evidence is uncertain, and new research may change policy or practice decisions, which is the case for drug use during pregnancy.

The unprecedented acceleration of research and production of new results requires a change in the ecosystem of production and dissemination of syntheses in order for research efforts to have an appropriate impact on public health decisions and medical practice and not remain dead letter, passing unnoticed because they are drowned in the masses [1, 19, 20]. For that, health care providers and decision-makers need to have access to up-to-date evidence syntheses with integration of all available evidence in a comprehensive, meaningful, and time-efficient manner. Currently, nothing like this is available in relation to the risk of medicine use during pregnancy.

The metaPreg project was set up in 2018 as an attempt to provide a solution to this situation. It is an initiative to carry out large-scale living evidence syntheses dedicated to the risk of drug use during pregnancy. In the long term, the MetaPreg project aims to cover all pharmacological treatments and to maintain up-to-date results in real-time. To make such a project feasible, two technologies have been developed: (i) an integrated infrastructure allowing a semi-automated realization of the whole synthesis process (study search, selection, extraction, etc.) (the semi-automation has been described elsewhere [21]) and (ii) an online open access platform (www.metaPreg.org) for real-time generation and optimized dissemination of the produced evidence syntheses.

This full-scale approach also ensures that all treatments are covered similarly, using a standard method developed to ensure this homogeneity. This article reports the master protocol that defines the general method that will be applied to produce the synthesis of all drugs. A subprotocol, deriving from this master protocol, could be established in order to take into account drugs or therapeutic classes specificities (such as long half-life, class effect, route of administration) and address particular questions (such as dose-effect relationship).

### Objective

To assess the risk for the fetus, the newborn, the infant, and the mother of a drug or therapeutic class used during pregnancy by synthesizing the available evidence derived from controlled observational studies and randomized controlled trials (RCTs).

## Methods and design

This systematic review and meta-analysis protocol are reported in accordance with the Preferred Reporting Items for Systematic review and Meta-Analysis Protocols (PRISMA-P) reporting guidelines [22] (see Additional file 1).

### Criteria for considering studies

#### Types of studies

Eligible studies will be studies reporting specific data for pregnancy outcomes after in utero exposure to the considered drug(s) with a comparator group (see section “dealing with multiple comparator groups” for more details).

Prospective cohort studies, historical cohort studies (also known as retrospective cohort studies), case-control studies, and, possibly, randomized clinical trials will be included. Studies will be included regardless of publication status or language of publication. Regarding languages other than French or English, machine translation tools will be used to confirm the eligibility of the study. Then, in case of inclusion, firstly we will call on researchers and/or doctors available in our international environment (i.e medical school with international scope) to read and ensure the translation of these publications, and if needed, a translation service will be solicited. We will assess all potentially relevant published articles and abstracts for inclusion. Information from on-going studies and interim analyses will be included.

We will exclude studies with inappropriate design (case reports, case series, cross-sectional studies, disproportionate analysis, adverse drug reactions reports…), studies without original data (review, letter to editor, comments…), or animal studies. Observational studies not presenting quantitative results (e.g., odds ratio, hazard ratio, relative risks, 95% confidence intervals, numbers of cases/population, observed and expected cases) or sufficient data to calculate treatment effect will also be excluded.

Only the most recent publication of iterative analyses on the same database will be included and in case of overlapping studies, only the one with a larger sample size will be kept or otherwise, with a methodology that provides a better consideration of the confounding factors (completeness of adjustment).

#### Types of outcomes

All the potential adverse pregnancy outcomes will be systematically considered without distinction between primary or secondary outcomes:

- Intrauterine deaths, such as early intrauterine death (spontaneous abortions, miscarriages), late fetal death (stillbirth), ectopic pregnancy, therapeutic termination of pregnancy, elective termination of pregnancy
- Major congenital malformations (MCM), as coded by the European Surveillance of Congenital Anomalies (EUROCAT): as a whole (all MCM), by malformation subgroups (e.g., congenital heart defects) or individually (e.g., atrial septal defect). Malformations not registered or excluded by EUROCAT will be not considered, except the whole group of minor malformations
- Growth parameters and prematurity, such as small for gestational age, macrosomia, low birthweight
- Neonatal disorders, as a whole, but also individually such as the Apgar score, need for neonatal medical care, persistent pulmonary hypertension, withdrawal syndrome
- Neurodevelopmental disorders, as a whole, but also individually such as autism spectrum disorder, attention deficit with or without hyperactivity disorder, cognitive delay, language disorder/delay, psychomotor disorder/delay
- Other long-term consequences, such as cancer, infant asthma, emotional disorders
- Maternal consequences, as a whole, but also individually, such as *postpartum* hemorrhage, gestational diabetes, pre-eclampsia, and placenta previa

For the neuro-developmental disorders, due to the diversity of measurement tools or diagnostic scales used in the studies, experts were consulted to produce the connection between the endpoints as reported in the included studies and the corresponding clinical entities (see Additional file 2).

### Search methods for identification of studies

Relevant studies will be identified in different electronic databases which have shown good coverage in health sciences [23, 24] (i.e PubMed/Medline and and EMBASE), from inception and without date restrictions, to ensure that all relevant literature is identified.

A specific search strategy will be developed for each molecule built on combinations of two categories of search terms: the search terms dedicated to the treatment (class or molecule) and those needed to identify analytical studies of consequences of in utero exposure to drugs. Search strategy templates are listed in Additional file 3.

A search based on therapeutic class will be adopted to improve exhaustivity. Indeed, some studies address the question at the class level and not at the level of a specific molecule. However, molecule-specific results may be reported in this kind of publication. Due to their global focus, however, these studies will not be identified by a molecule-specific search equation. The search must be extended to the class to identify them. To avoid duplicate searches of the same class for each molecule, a single search will be performed integrating the class and all the specific molecules it includes. The references reported by this global search will then be dispatched among the different systematic reviews concerning the molecules.

Relevant studies will also be identified through a snowballing approach to identify relevant papers based on the reference lists of published meta-analyses and/or systematic reviews. Additional efforts will be performed to identify abstracts and presentations made at appropriate conferences and clinical trial databases.

### Selection of studies

A two-step process will be utilized for the study selection. The first step will be a selection using title and abstract of bibliographic records of all found references. Abstracts of all studies identified in the above search will be screened by one biocurator (a scientist who extracts data from a variety of sources to standardize them and make it machine readable, more discoverable, and accessible to the public [25]) who will receive a dedicated training program before joining the team, and assisted by proprietary automation tools based on artificial intelligence [21].

For the second step, we will obtain the full-text reports of studies that are potentially relevant. Studies under consideration will be assessed for whether they fulfill the inclusion criteria and methodological design without regard to their results.

For each step, in case of doubt about inclusion of a study, the matter will be discussed with the scientific directors of the project until agreement is reached. The feasibility and acceptance of this semi-automated process was assessed and it was shown that it reduces the time required for a meta-analysis without altering the reliability in the study selection, and more globally in the expert confidence in the methodological and scientific rigor [21]. The process of study selection will be documented.

### Data extraction and data collection process

All studies that meet the inclusion criteria at the full-text phase will have the following data extracted (where available) and recorded into our production platform.

The following information will be collected:

- Study description: information on first author, year of publication, main outcome, country of study, source of data, study period, population description, exposure definition, non-exposure definition, type of control, case description, control description, and sample size
- Method: type of study, exposition measure, outcome measure, follow-up period, and confounding factors that were taken into consideration
- Results: for each dichotomous adverse outcome, we will extract the number of events, the number of total participants in each study group, the maximally adjusted (regarding a pre-established list of potential confounders for each class of endpoint, see Additional file 4) relative risk treatment effect (as reported in the paper, odds ratio, risk ratio, hazard ratio or standardized incidence (rate)/mortality ratio/relative risk), and 95% confidence intervals, exposition period, and nature of the control group.

Data collection will be performed by one biocurator and checked by a second one during a quality control process where every item, including missed details, will be checked. This quality control will be completed by a cross-check with previously published reviews and meta-analysis. Detected discrepancies will be discussed by the biocurators until resolution or during a project meeting.

### Risk of bias assessment

A risk of bias of included studies will be assessed at an outcome level using the Cochrane Risk of Bias Tool for Non-Randomized Studies of Interventions (ROBINS-I) for the observational studies and Risk of Bias-2 (ROB-2) for the RCTs.

The ROBINS-I tool signaling questions were adapted to observational studies evaluating medicine safety in pregnancy. Six types of bias will be considered: (a) selection bias, (b) confusion bias, (c) bias in classification of exposure, (d) bias due to missing data, (e) bias in measurement outcomes, and (f) bias in selection of reported results. We considered that the ROBINS-I item on bias risk at intervention was too specific to efficacy study, and we will not apply it to purely safety studies.

For bias due to confounding four levels of risk will be considered: low, moderate, serious, and critical. For other biases, only three levels of risk were considered (low, moderate, and critical), as we did not identify situations where a degree of fineness between critical and serious can be distinguished.

The findings of the assessment of RCT with the ROB-2 will be reported by using the corresponding bias dimension of the ROBINS-I using the table of correspondence given in the ROBINS-I paper.

### Data synthesis and analysis

The meta-analysis will be performed by using only the summary data. No attempt will be done to obtain the individual patient data.

Given the need to control for confounding factors in observational studies, we will use adjusted measures as the primary effect measures when reported by the authors. Concerning adjustment strategies, we will specify which confounding factors have been considered in the study design and analysis. If no adjusted measures were given as part of the primary analysis, we will use unadjusted measures.

If data will be available for unadjusted dichotomous outcomes, we will calculate the odds ratio (OR) with 95% confidence interval. Data from primary observational studies will be used to perform random-effects meta-analyses.

Relative treatment effect as reported will be used to estimate the summary effect size and its 95% confidence interval using the inverse variance method based on the DerSimonian and Laird random effects model.

#### Dealing with multiple comparator groups

Studies could consider different comparator groups (i.e., unexposed-sick; unexposed disease-free or general population or not otherwise specified; and sick exposed to other treatments) and thus report as many estimates of the treatment effect. Only one estimate will be used for the meta-analysis. As “untreated sick comparator” takes into consideration the potential impact of the disease, estimates using this control will be preferentially used for the primary analysis.

In the primary analysis, control groups will be chosen in the following order of preference:

- “Unexposed, sick”, i.e., women with the same disease, who do not receive the drug of interest (nor an alternative treatment for the concerned disease) during pregnancy, but that could have been received treatment prior to pregnancy
- “Unexposed, not otherwise specified or general population,” i.e., women not exposed to the drug of interest, without precision of the illness status
- “Unexposed, disease free,” i.e., women without the same disease, not exposed to the drug of interest
- “Exposed to other treatment, sick,” i.e., women with the same disease, exposed to a different drug

Moreover, a subgroup analysis using all included studies and according to different group controls will be available.

#### Dealing with period of exposure

Period exposition will be classified according to the following predefined categories:

- “During pregnancy” (anytime or not otherwise specified)
- “Throughout pregnancy” (i.e., all along the pregnancy)
- “Early pregnancy,” “1st trimester,” “at least 1st trimester,” and “1st and 2nd trimester”
- “2nd trimester”, “3rd trimester”, “2nd and/or 3rd trimester”, and “late pregnancy” (that corresponds to the period of organs maturation)
- “Days before delivery” (i.e., the period right before delivery)

Only data from the period of exposure relevant for a specific outcome will be used. For studies that consider different periods of exposure for the same outcome, we will use the estimate corresponding to the most relevant period of exposure according to the outcome.

The list of relevant periods of exposure for the various endpoints was established in collaboration with an expert of this domain (see Additional file 5 for more details). For instance, only exposure during early pregnancy or first trimester will be included for the malformations; only exposure during the late pregnancy will be included for neonatal withdrawal.

#### Dealing with zeros

In case of zero number of events in one group, a continuity correction will be used by replacing the zero by 0.5 (equivalent to arguments incr=0.5, allincr=F, addincr=F in metabin function of meta package in R).

#### Meta-biases control and assessment

Publication bias and small study effect will be assessed by inspecting visually the funnel plot for asymmetry and with Egger’s test for funnel plot including ten studies or more.

#### Assessment of heterogeneity

We will assess heterogeneity of treatment [26] effect visually from the forest plot. This will help determine whether the differences between the results of studies are greater than would be expected by chance alone. We will also assess heterogeneity by means of the I-squared statistic [27]. The random-effects model is selected a priori to synthesize the epidemiological data, as it considers both within-study and between-study variation by incorporating the heterogeneity of effects into the overall analyses.

#### Sensitivity analyses

Due to the potential heterogeneity in the set of studies, w e will undertake sensitivity analyses to determine the robustness of the meta-analysis results. We will group the studies (if a sufficient number of studies exist) according to the following:

- Type of control groups (unexposed sick, unexposed not-sick, exposed to other treatments)
- Study design (cohort studies, case-control studies, randomized controlled trials)

#### Handling of overlapping/updated data

For studies published in multiple articles, reports, or presentations, double-counting of data will be avoided by ensuring that the sample in a given study does not overlap with the samples included in any other study. To identify these situations, we have developed a tool which detects potential overlaps, i.e studies using the same data source to study the same outcomes and then we will examine in depth study (notably study period, definition of the study population, …) to confirm or not the overlapping. If overlap appears, data from the most recent or most comprehensive paper or with a higher number of exposed subjects will be retained.

#### Dealing with missing data

No attempts will be made to contact study authors to obtain missing data (e.g., adjusted results, participants, intervention, or outcome details). Loss to follow-up will be reported and assessed as a potential source of bias in our risk of bias assessment.

### Assessment of the certainty of the evidence

Any global assessment of the certainty of the evidence will be reported on the dissemination platform given the observational nature of studies in this field. All results are expected to be of very-low certainty by GRADE (Grading of Recommendations, Assessment, Development and Evaluation), due to risk of bias mainly and inconsistency. To help the users to determine on their own the level of confidence they have in the results they are browsing, the risk of bias on each dimension of the ROBINS-I will be reported for all studies documenting each outcome.

### Living systematic review approach

The literature searches for these living reviews will be updated continuously on a weekly basis using software robots screening automatically RSS feeds of relevant journals and other sources likely to report news about pregnancy study. These robots based on machine learning were trained to detect analytical studies about drugs during pregnancy. Study analysis and data extraction will be performed immediately, and a corresponding systematic review will be updated. Due to the dynamic nature of our dissemination platform, information and evidence synthesis available on the metaPreg website will be thus instantaneously updated as soon as new pieces of evidence are available.

### Availability of data and materials

All the data used and analyzed are available on the metaPreg.org website.

### Amendments

If any amendments to this master protocol are necessary, the date and specific changes to the protocol will be traced, with their rationale, on the online master protocol downloadable on www.metapreg.org website.

## Discussion

The living meta-analysis approach proposed by the metaPreg project will enable direct access to synthesized results regarding the risk of drugs during pregnancy for which relevant epidemiological studies are available.

The proposed project has a number of strengths. All evidence syntheses will be standardized following the methods previously described. Furthermore, the meta-analyses will be continuously updated as new results become available. The dissemination of the results will be done using an online tool facilitating the exploration and visualization of the mass of information produced.

The achievement of a project of this magnitude is only possible through a semi-automated realization of these systematic reviews and the professionalization of a team on this topic. This approach represents the application to the synthesis of clinical data of the principle of biocuration developed for the constitution of large knowledge bases gathering the results of studies on the genome for example.

The semi-automation approach employed by metaPreg has been described elsewhere, and the study showed that semi-automated meta-analyses improve completeness, save time without altering expert confidence or losing the methodological quality required by meta-analyses. Indeed, typically, the time to completion was 14 working days with metaPreg versus 24.7 with the conventional meta-analysis system, with a higher number of included studies (39 for metaPreg versus 24 in the conventional meta-analysis, respectively) and a higher number of outcomes integrated in metaPreg (28 for metaPreg versus 16 in the conventional meta-analysis, respectively) [21]. This process has also been used for different publications [28, 29].

Our planned living systematic review and meta-analysis are not without limitations. The included studies are subject to numerous biases. Except in exceptional cases, it is often impossible to identify a group of studies of sound methodological quality, making it impossible to restrict the meta-analysis to the best studies.

Most studies are exploratory and performed on secondary data, which can lead to p-hacking (also known as data fishing) and selective publication of results. A publication bias in favor of overdetection of risks is therefore possible. Whenever the number of studies allows, the possibility of publication bias will be systematically assessed.

Pregnant women are routinely excluded from randomized trials; therefore, clinical evaluation of the safety of drugs during pregnancy is very rarely performed before marketing. Therefore, epidemiological studies based on observational data are the main means of evaluating the impact of drugs on fetal development. These ones are conducted on a case-by-case basis, depending on the availability of data (i.e., exposed pregnancies accidentally or after medical prescription or after self-administration). Moreover, to date, the investigation of the risk of drugs during pregnancy has largely focused on birth defects (such as malformations) and very little on functional or long-term disorders (such as neurodevelopmental disorders). Therefore, it is impossible to systematically document all the risks of all drugs during pregnancy, but the absence of available data does not mean that the drugs are safe.

In conclusion, this approach, which combines the semi-automation of systematic reviews and meta-analysis with the biocuration approach, associated with an open-access web platform dissemination, should make it possible to overcome the limitations of traditional publication for the dissemination of up-to-date evidence syntheses concerning the risk of drugs during pregnancy.

## Supplementary Information

**Additional file 1.** PRIMA-P checklist.

**Additional file 2.** Mapping table used for neurodevelopmental disorders: neurodevelopmental measurement tools or diagnostic scales used in the included studies and their corresponding classification in the meta-analyses.

**Additional file 3.** Search strategies in MEDLINE and EMBASE.

**Additional file 4.** Pre-established list of potential confounders for each class of endpoints.

**Additional file 5.** Relevancy (Yes/No) of the period of exposures for each outcome. If the period of exposure is relevant (Yes), the results available in the publication for the considered outcome will be included in the meta-analysis, otherwise (No), the results available in the publication for the considered outcome will be excluded.

## Data Availability

Not applicable.

## Abbreviations

EUROCAT: European Surveillance of Congenital Anomalies
GRADE: Grading of Recommendations, Assessment, Development and Evaluation
MCM: Major congenital malformations
PRISMA-P: Preferred Reporting Items for Systematic review and Meta-Analysis Protocols
OR: Odd ratio
RCT: Randomized controlled trial
ROB-2: Risk of bias 2
ROBINS-I: Risk of Bias Tool for Non-Randomized Studies of Interventions

## References

[1] Boutron I, Créquit P, Williams H, Meerpohl J, Craig JC, Ravaud P (2020). Future of evidence ecosystem series: 1. introduction evidence synthesis ecosystem needs dramatic change. J Clin Epidemiol.

[2] Metelli S, Chaimani A (2020). Challenges in meta-analyses with observational studies. Evid Based Ment Health..

[3] Grzeskowiak LE, Gilbert AL, Morrison JL (2013). Methodological challenges in using routinely collected health data to investigate long-term effects of medication use during pregnancy. Ther Adv Drug Saf..

[4] Damase-Michel C. D1.2 Core evidence elements for generating medication safety evidence for pregnancy using population-based data. IMI2 821520 - ConcePTION Ref. Ares(2021)1481289 - 25/02/2021. 2021.

[5] Huybrechts KF, Bateman BT, Hernández-Díaz S (2019). Use of real-world evidence from healthcare utilization data to evaluate drug safety during pregnancy. Pharmacoepidemiol Drug Saf..

[6] Wood ME, Lapane KL, Gelder MMHJ, Rai D, Nordeng HME (2018). Making fair comparisons in pregnancy medication safety studies: an overview of advanced methods for confounding control. Pharmacoepidemiol Drug Saf..

[7] Morales DR, Slattery J, Evans S, Kurz X. Antidepressant use during pregnancy and risk of autism spectrum disorder and attention deficit hyperactivity disorder: systematic review of observational studies and methodological considerations. BMC Med. 2018 [cited 2022 May 23];16. Available from: https://bmcmedicine.biomedcentral.com/articles/10.1186/s12916-017-0993-3. 10.1186/s12916-017-0993-3 PMC5767968 29332605

[8] Lee YH (2018). An overview of meta-analysis for clinicians. Korean J Intern Med..

[9] Lunny C, Reid EK, Neelakant T, Chen A, Zhang JH, Shinger G (2022). A new taxonomy was developed for overlap across “overviews of systematic reviews”: a meta-research study of research waste. Res Synth Methods..

[10] Ioannidis JPA (2016). The mass production of redundant, misleading, and conflicted systematic reviews and meta-analyses: mass production of systematic reviews and meta-analyses. Milbank Q..

[11] Chapelle C, Ollier E, Girard P, Frere C, Mismetti P, Cucherat M (2021). An epidemic of redundant meta-analyses. J Thromb Haemost..

[12] Andersen MZ, Fonnes S, Andresen K, Rosenberg J (2021). Most published meta-analyses were made available within two years of protocol registration. Eur J Integr Med..

[13] Borah R, Brown AW, Capers PL, Kaiser KA (2017). Analysis of the time and workers needed to conduct systematic reviews of medical interventions using data from the PROSPERO registry. BMJ Open..

[14] Bashir R, Surian D, Dunn AG (2018). Time-to-update of systematic reviews relative to the availability of new evidence. Syst Rev..

[15] Thomas J, Noel-Storr A, Marshall I, Wallace B, McDonald S, Mavergames C (2017). Living systematic reviews: 2. combining human and machine effort. J Clin Epidemiol.

[16] Amezcua-Prieto C, Fernández-Luna JM, Huete-Guadix JF, Bueno-Cavanillas A, Khan KS (2020). Artificial intelligence and automation of systematic reviews in women’s health. Curr Opin Obstet Gynecol..

[17] Marshall IJ, Wallace BC. Toward systematic review automation: a practical guide to using machine learning tools in research synthesis. Syst Rev. 2019;8:163, s13643-019-1074–9. 10.1186/s13643-019-1074-9 PMC6621996 31296265

[18] Clark J, Glasziou P, Del Mar C, Bannach-Brown A, Stehlik P, Scott AM (2020). A full systematic review was completed in 2 weeks using automation tools: a case study. J Clin Epidemiol..

[19] Elliott JH, Synnot A, Turner T, Simmonds M, Akl EA, McDonald S (2017). Living systematic review: 1. introduction—the why, what, when, and how. J Clin Epidemiol.

[20] Akl EA, Meerpohl JJ, Elliott J, Kahale LA, Schünemann HJ, Agoritsas T (2017). Living systematic reviews: 4. living guideline recommendations.. J Clin Epidemiol.

[21] Ajiji P, Cottin J, Picot C, Uzunali A, Ripoche E, Cucherat M, et al. Feasibility study and evaluation of expert opinion on the semi-automated meta-analysis and the conventional meta-analysis. Eur J Clin Pharmacol. 2022 [cited 2022 May 23]; Available from: https://link.springer.com/10.1007/s00228-022-03329-8. 10.1007/s00228-022-03329-8 35501476

[22] Shamseer L, Moher D, Clarke M, Ghersi D, Liberati A, Petticrew M (2015). Preferred reporting items for systematic review and meta-analysis protocols (PRISMA-P) 2015: elaboration and explanation. BMJ..

[23] Frandsen TF, Eriksen MB, Hammer DMG, Christensen JB, Wallin JA (2021). Using Embase as a supplement to PubMed in Cochrane reviews differed across fields. J Clin Epidemiol..

[24] Hartling L, Featherstone R, Nuspl M, Shave K, Dryden DM, Vandermeer B (2016). The contribution of databases to the results of systematic reviews: a cross-sectional study. BMC Med Res Methodol..

[25] International Society for Biocuration (2018). Biocuration: distilling data into knowledge. PLOS Biol..

[26] Higgins JPT, Thomas J, Chandler J, Cumpston M, Li T, Page MJ, et al. Cochrane Handbook for Systematic Reviews of Interventions version 6.3 (updated February 2022). Cochrane; 2022. Available from: Available from www.training.cochrane.org/handbook.

[27] Higgins JPT (2003). Measuring inconsistency in meta-analyses. BMJ..

[28] Auffret M, Cottin J, Vial T, Cucherat M (2019). Clomiphene citrate and neural tube defects: a meta-analysis of controlled observational studies. BJOG Int J Obstet Gynaecol..

[29] Picot C, Berard A, Grenet G, Ripoche E, Cucherat M, Cottin J (2020). Risk of malformation after ondansetron in pregnancy: an updated systematic review and meta-analysis. Birth Defects Res..

