# Supplementary material for: Risk of drug use during pregnancy: master protocol for living systematic reviews and meta-analyses performed in the metaPreg project
Source: Syst Rev. 2023 Jun 21;12:101. doi: 10.1186/s13643-023-02256-8 (PMC10286473; doi:10.1186/s13643-023-02256-8)
Supplement: Supplementary file 2 — Additional file 2. Mapping table used for neurodevelopmental disorders: neurodevelopmental measurement tools or diagnostic scales used in the included studies and their corresponding classification in the meta-analyses. [file 13643_2023_2256_MOESM2_ESM.pdf]

Additional file 2: Table of correspondence used for neurodevelopmental disorders: neurodevelopmental measurement tools or diagnostic scales used in the included studies and their corresponding classification in the meta-analyses (V2)

**Adverse outcome**	**Definitions of the criteria and measurement tools that can be used for this dimension**
NEURODEVELOPMENTAL DISORDERS	
**Neurodevelopmental disorders (without differentiation by authors)**	1. Neurodevelopmental disorders, without differentiation by authors, as recorded in medical record **(ICD-10 F70-F90.9 or clinical diagnosis)**: several cognitive disorders (e.g. cognitive delay, speech therapy, ...) **and** at least one behavioral disorder (ADHD, ASD, …)<br>2. Additional/special educational needs…
**Cognitive developmental disorders – infants** (< 3 years old)	1. Diagnosis of Developmental disorder or delay recorded in medical record (if possible, established or confirmed by a developmental paediatrician or paediatric neurologist)<br>2. Scores of global Developmental Quotient (DQ) **< 85 or < 1 SD,** assessed with:<br>○ Griffiths mental development scales (Griffiths)<br>○ Bayley Scales of Infant Development (Bayley) (*corrected for number of weeks born prematurely*)<br>○ Denver Developmental Screening Test (DDST)<br>○ Wechsler Preschool and Primary Scale of Intelligence (WPPSI)<br>○ Non-verbal intelligence test of Snijders-Oomen (SON-R) (*ok for deaf children or those with hearing difficulties as well as for valid children*)<br>○ The Revision of the Amsterdam Children's Intelligence Test (RAKIT)<br>○ Early childhood development scale of Brunet-Lézine<br>○ Mullen Scales of Early Learning<br>3. Mental Development Index or score of performance of Intellectual Quotient (IQ) < 85 (or < 1 SD), assessed by global scales, as:<br>○ Bayley Scales of Infant Development (Bayley)<br>○ Griffiths mental development scales (Griffiths)<br>○ Wechsler Preschool and Primary Scale of Intelligence (WPPSI)<br>4. Overall score < 85 (or < 1 SD) on an Adaptive Behavior scale (with homogeneity in the scale's domains), as:<br>○ VINELAND-II - Vineland adaptive behavior scale (or Vineland Social Maturity Scale – old name)<br>○ ABAS II (Adaptive behavior assessment system) (parents and/or teachers)
**Cognitive developmental disorders – children** (3-6 years old)<br>**Cognitive developmental disorders – children** (> 6 years old)	1. Diagnosis of Developmental disorder or delay recorded in medical record (if possible, established or confirmed by a developmental paediatrician or paediatric neurologist)<br>2. Scores of global Intellectual Quotient (IQ) **< 85 or < 1 SD,** assessed with:<br>○ Wechsler Intelligence Scale for Children (WISC)<br>○ Wechsler Preschool and Primary Scale of Intelligence (WPPSI)<br>○ Differential ability scales (DAS)<br>○ Non-verbal intelligence test of Snijders-Oomen (SON-R) (ok for deaf children or those with hearing difficulties as well as for valid children)<br>○ The Revision of the Amsterdam Children's Intelligence Test (RAKIT)<br>○ Color Progressive Matrices (CPM or PM47)<br>○ Color Progressive Matrices (CPM-BF)<br>○ Standard Progressive Matrices (SPM or PM38)<br>○ Standford-Binet intelligence scales<br>○ McCarthy Scales of Children's Abilities<br>3. Mental Development Index or score of performance of Intellectual Quotient (IQ) < 85 (or < 1 SD), assessed by global scales, as:<br>○ Griffiths mental development scales (Griffiths)<br>○ Wechsler Preschool and Primary Scale of Intelligence (WPPSI)<br>4. Overall score < 85 (or < 1 SD) on an Adaptive Behavior scale (with homogeneity in the scale's domains), as:<br>○ VINELAND-II - Vineland adaptive behavior scale (or Vineland Social Maturity Scale – old name)<br>○ ABAS II (Adaptive behavior assessment system) (parents and/or teachers)

Adverse outcome	Definitions of the criteria and measurement tools that can be used for this dimension
**Severe cognitive developmental delay (Mental retardation) – infants**<br>(< 3 years old)	1. Diagnosis of **severe** cognitive developmental delay recorded in medical record **(ICD-9: 317-319; ICD-10: F70-F79 or clinical diagnosis)** (if possible, established or confirmed by developmental a paediatrician or paediatric neurologist)<br>2. Scores of global Developmental Quotient (DQ) **< 70 or < 2 SD** (higher degree of severity) assessed with:<br>○ Griffiths mental development scales (Griffiths)<br>○ Bayley Scales of Infant Development (Bayley) *(corrected for number of weeks born prematurely)*<br>○ Denver Developmental Screening Test (DDST)<br>○ Wechsler Preschool and Primary Scale of Intelligence (WPPSI)<br>○ Non-verbal intelligence test of Snijders-Oomen (SON-R) *(ok for deaf children or those with hearing difficulties as well as for valid children)*<br>○ the Revision of the Amsterdam Children's Intelligence Test (RAKIT)<br>○ Differential ability scales (DAS)<br>○ Early childhood development scale of Brunet-Lézine<br>3. Overall score < 70 (or < 2 SD) on an Adaptive Behavior scale (with homogeneity in the scale's domains), as:<br>○ VINELAND-II - Vineland adaptive behavior scale (or Vineland Social Maturity Scale – old name)<br>○ ABAS II (Adaptive behavior assessment system) (parents and/or teachers))<br>4. Severe learning disorders (school results…), with indication of global cognitive delay
**Severe cognitive developmental delay (Mental retardation) – children**<br>(3-6 years old)<br><br>**Severe cognitive developmental delay (Mental retardation) – children**<br>(> 6 years old)	1. Diagnosis of **severe** cognitive developmental delay recorded in medical record **(ICD-9: 317-319; ICD-10: F70-F79 or clinical diagnosis)** (if possible, established or confirmed by developmental a paediatrician or paediatric neurologist)<br>2. Scores of global Intellectual Quotient (IQ) **< 70 or < 2 SD** (higher degree of severity) assessed with:<br>○ Griffiths mental development scales (Griffiths)<br>○ Bayley Scales of Infant Development (Bayley)<br>○ Denver Developmental Screening Test (DDST)<br>○ Wechsler Preschool and Primary Scale of Intelligence (WPPSI) before 5 years old<br>○ Wechsler Intelligence Scale for Children (WISC) after 5 years old<br>○ Wechsler Adult Intelligence Scale (WAIS) after 16 years old<br>○ Non-verbal intelligence test of Snijders-Oomen (SON-R) *(ok for deaf children or those with hearing difficulties as well as for valid children)*<br>○ the Revision of the Amsterdam Children's Intelligence Test (RAKIT)<br>○ Differential ability scales (DAS)<br>3. Overall score < 70 (or < 2 SD) on an Adaptive Behavior scale (with homogeneity in the scale's domains), as:<br>○ VINELAND-II - Vineland adaptive behavior scale (or Vineland Social Maturity Scale – old name)<br>○ ABAS II (Adaptive behavior assessment system) (parents and/or teachers)<br>4. Severe learning disorders (school results…), with indication of global cognitive delay
**Language disorders or delay**<br>(whatever age)	1. Diagnosis of language disorder or delay recorded in medical record **(ICD-10: F80 or clinical diagnosis)** (if possible established or confirmed by a developmental paediatrician or paediatric neurologist)<br>2. Dysphasia (ICD-10: F80 ; DSM-5: 315.39 ; or clinical diagnosis)<br>3. Verbal Intellectual Quotient (IQ) < 85 or < 1 SD, or language disorder or delay assessed with:<br>○ The Reynell Developmental Language Scale<br>○ Clinical evaluation of language fundamentals (CELF)<br>○ Learning Accomplishment Profile<br>○ Subset "Hearing and Language" in Griffiths scales<br>○ Subset verbal of IQ or DQ<br>○ Subset Communication VINELAND<br>○ Communication and Symbolic Behavior Scales Developmental Profile (CSBS-DP)

Adverse outcome	Definitions of the criteria and measurement tools that can be used for this dimension
	○ Comprehensive Language Assessment<br>○ the Phelps Kindergarten Readiness Scale II (if no other test assesses language)<br>○ (Peabody Picture Vocabulary Test; Receptive Expressive Emergent Language Scale; Expressive One Word Picture Vocabulary Test or Sequenced Inventory of Communication Development)<br>4. Medical record: Need for speech therapy, with indication for oral language disorders, if possible
**Learning disorders** (whatever age)	1. Diagnosis of learning disorder recorded in medical record **(ICD-10: F81; DSM-5:315 except 315.4; or clinical diagnosis)** (if possible established or confirmed by a developmental paediatrician or paediatric neurologist)<br>2. Dyslexia, dysorthographia, … (*except dysphasia and dyspraxia*) **(ICD-10: F82; DSM-5: 315.0, 315.1, 315.2; or clinical diagnosis)**<br>3. Academic performances (spelling, reading, math, …): low success in exams, lower grades…
**Psychomotor developmental disorders or delay** (whatever age)	1. Diagnosis of psychomotor disorder or delay or diagnosis of neuromotor deficit recorded in medical record **(ICD-10: F82; DSM-5: 315.4; or clinical diagnosis)** (if possible established or confirmed by a trained practitioner); confirmed delay in motor development milestones (infant failing to sit by 10 months old or walk by 18 months old …)<br>2. Dyspraxia **(ICD-10: F82; DSM:315.4; or clinical diagnosis)**<br>3. Psychomotor Intellectual Quotient **< 85 (or < 1 SD)** or motor disorders, assessed with:<br>○ Miller function and participation scales (M-FUN)<br>○ Psychomotor subsets of global scales:<br>■ Griffiths scales<br>■ BSID II (Bayley Scales of Infant Development, motor scale)<br>■ Schedule of Growing Skills II (SGS II) (Locomotion = gross motor ; Handling = Fine motor)<br>○ Motor VINELAND<br>○ Touwen's test<br>○ The Alberta Infant Motor Scale (AIMS)
**Risk of ASD (Autism spectrum disorder)** (whatever age)	1. Risk of ASD **(< 18 months old)**, assessed with:<br>○ Checklist for Autism in Toddlers (CHAT)<br>○ Modified Checklist Autism for Toddlers (M-CHAT)<br>○ Modified Checklist Autism for Toddlers Révisé/Follow- up (M-CHAT-R/F)<br>○ Quantitative Checklist for Autism (Q-CHAT)<br>2. Risk of ASD **(>18 months old)**, assessed with:<br>○ The 40-item Social Communication Questionnaire (SCQ)<br>○ Modified Autism Spectrum Screening Questionnaire (ASSQ)<br>○ Autism Screening Questionnaire (ASQ ; ASQ-II)<br>○ Autism- spectrum Quotient (AQ)<br>○ Childhood Autism Rating Scale (CARS) when score between 27-29<br>○ Social Responsiveness Scale (SRS)<br>○ Social Emotional Questionnaire
**Diagnosis of ASD (Autism spectrum disorder)** (whatever age)	1. Diagnosis of ASD or subtype of ASD (Childhood autism, Atypical autism, Rett syndrome, Other childhood disintegrative disorder, Overactive disorder associated with mental retardation and stereotyped movements, Asperger syndrome, Other pervasive developmental disorders or unspecified), recorded in medical record **(ICD-9: 299; ICD-10: F84.0-F84.9 ; DSM-5: 299 ; or clinical diagnosis)** (if possible established or confirmed diagnosis in hospital/medical records made by a paediatrician or child psychiatrist)<br>2. Diagnosis of ASD (global) assessed with:<br>○ Childhood Autism Rating Scale (CARS) when score >30<br>○ Autism Diagnostic Observation Schedule (ADOS)<br>○ Revised Behavior Summarized Evaluation scale (BSE-R)<br>○ Autism Diagnostic Interview (ADI)<br>○ Autism Diagnostic Interview – revised (ADI-R)

Adverse outcome	Definitions of the criteria and measurement tools that can be used for this dimension
**Diagnosis of ASD (Autism spectrum disorder) or Risk (when Diagnostic unavailable)**	See the categories of ASD above.
**Risk of ADHD (Attention deficit hyperactivity disorder)** (whatever age)	1. Risk of ADHD, whatever age but assessed by **one investigator with**:<br>○ Conners' rating scales (parents or teachers)<br>○ Attention Problems and Hyperactivity Scales ex: Strengths and Difficulties Questionnaire (SDQ); Behavior assessment system for children (BASC) (parents or teachers)<br>○ Child Behaviour Checklist (CBCL)<br>○ Social Emotional Questionnaire
**Diagnosis of ADHD (Attention deficit hyperactivity disorder)** (whatever age)	1. Diagnosis of ADHD recorded in medical record **(ICD-10: F90; DSM-5: 314 ; or clinical diagnosis)** (if possible established or confirmed by a paediatrician or child psychiatrist)<br>2. Prescription of a treatment for ADHD (methylphenidate, …)<br>3. ADHD, assessed by **several investigators (≥2)**<br>○ Conners' rating scales (parents and teachers)<br>○ Behavior assessment system for children (BASC) (parents and teachers)<br>○ Attention Problems and Hyperactivity Scales<br>○ Child Behaviour Checklist
**Diagnosis of ADHD (Attention deficit hyperactivity disorder) or Risk (when Diagnostic unavailable)**	See the categories of ADHD above.
PSYCHIATRIC AND PSYCHOLOGICAL DISORDERS OF THE CHILD	
**Behavioral disorders**	1. Diagnosis (**ICD10: F91;92**) reported in a Medical record: DSM-IV or DSM-V or ICD or confirmed diagnosis in hospital/medical records made by a paediatrician or child psychiatrist<br>2. Child Behavior Checklist (CBCL) - Externalized disorders (oppositional disorders, « aggressive symptoms », …) (Subclinic: t-scores > 60-65 %)<br>3. Behavior assessment system for children (BASC) (aggression, …)
**Emotional disorders**	1. Diagnosis of depression, anxiety, … reported in a Medical record: DSM-IV or DSM-V or ICD (ICD-10 codes **F40-F48** (anxiety); **F30-F39**; **F93** (anxiety of child) confirmed diagnosis in hospital/medical records made by a paediatrician or child psychiatrist.<br>2. Child Behavior Checklist (CBCL) - Internalized disorders (Child affective problems, anxiety problems, …) (Subclinic: t-scores > 60-65 %)<br>3. Behavior assessment system for children (BASC) (depression ou anxiety)

DQ: Developmental quotient; IQ: Intellectual quotient; ICD: International Classification of Diseases; DSM: Diagnostic and Statistical Manual of Mental Disorders; ADHD: Attention deficit with or without hyperactivity disorder; ASD: Autism spectrum disorder
