# Supplementary material for: Risk of drug use during pregnancy: master protocol for living systematic reviews and meta-analyses performed in the metaPreg project
Source: Syst Rev. 2023 Jun 21;12:101. doi: 10.1186/s13643-023-02256-8 (PMC10286473; doi:10.1186/s13643-023-02256-8)
Supplement: Supplementary file 3 — Additional file 3. Search strategies in MEDLINE and EMBASE. [file 13643_2023_2256_MOESM3_ESM.pdf]

Additional file 3: Search strategies

The search strategy for the treatment will include the terms (MESH or EMTREE, pharmacological action and words) corresponding to the therapeutic or pharmacological class as well as all the terms corresponding to the molecules belonging to this class.

PUBMED

(<<<treatment search strategy>>>) AND ("birth defects" OR "birth defects-drug exposure" OR "teratogenic risk" OR "teratogenicity" OR "prenatal exposure" OR "prenatally exposed" OR "fetal exposure" OR "congenital anomaly" OR "fetal anomalies" OR "congenital anomalies" OR "congenital malformation" OR "congenital malformations" OR "congenital major malformations" OR "congenital disorders" OR "cardiovascular defects" OR "preterm birth" OR "stillbirth" OR "miscarriage" OR "spontaneous abortion" OR "use during pregnancy" OR "exposure in pregnancy" OR "exposure during pregnancy" OR "exposed in utero" OR "first-trimester exposure" OR Teratogens OR "Birth defect"[Mesh] OR "Congenital Abnormalities"[Mesh] OR "Fetal Death/chemically induced"[Mesh] OR "Fetal Development/drug effects"[Mesh] OR "Fetal Diseases/chemically induced"[Mesh] OR "Fetus/drug effects"[Mesh] OR "Stillbirth"[Mesh] OR "Teratogens"[Mesh] OR "Abortion, Spontaneous"[MESH]) AND ("cohort study" OR "prospective study" OR "prospective observational study" OR "case-control study" OR "prospective follow-up study" OR "prospective follow-up" OR "meta-analysis" OR "systematic review" OR "retrospective study" OR "registry" OR "birth register" OR "observational study" OR "population based health datasets" OR "population health data" OR "Cohort Studies"[MESH] OR "Prospective Studies"[Mesh] OR "Case-Control Studies"[Mesh] OR "Observational Study"[ptyp] OR "Meta-Analysis"[ptyp] OR "Longitudinal Studies"[MESH] OR "Registries"[MESH] OR "Retrospective Studies"[MESH] OR "Randomized Controlled Trial"[ptyp] OR "matched controls" OR "matched control" OR "case and control" OR "compared with controls" OR "case-control" OR "healthy controls" OR disproportionality OR "proportional reporting ratio" OR "odds ratio" OR "hazard ratio") `

EMBASE

(<<<treatment search strategy>>>) AND ('birth defect':de,ab,ti OR 'teratogenic risk':de,ab,ti OR 'prenatal exposure':de,ab,ti OR 'prenatally exposed':de,ab,ti OR 'fetal exposure':de,ab,ti OR 'congenital anomaly':de,ab,ti OR 'fetal anomalies':de,ab,ti OR 'congenital anomalies':de,ab,ti OR 'congenital malformation':de,ab,ti OR 'Congenital malformations':de,ab,ti OR 'congenital major malformations':de,ab,ti OR 'congenital disorders':de,ab,ti OR 'cardiovascular defects':de,ab,ti OR 'preterm birth':de,ab,ti OR stillbirth:de,ab,ti OR miscarriage:de,ab,ti OR 'spontaneous abortion':de,ab,ti OR 'use during pregnancy':de,ab,ti OR 'exposure in pregnancy':de,ab,ti OR 'exposure during pregnancy':de,ab,ti OR 'exposed in utero':de,ab,ti OR 'first-trimester exposure':de,ab,ti OR teratogen*:de,ab,ti OR 'drug induced malformation':de,ab,ti OR 'pregnancy outcome':de,ab,ti) AND ('cohort study':de,ab,ti OR 'Cohort Studies':de,ab,ti OR 'prospective study':de,ab,ti OR 'prospective observational study':de,ab,ti OR 'case-control study':de,ab,ti OR 'Case-Control Studies':de,ab,ti OR 'prospective follow-up study':de,ab,ti OR 'prospective follow-up':de,ab,ti OR 'Prospective Studies':de,ab,ti OR 'meta-analysis':de,ab,ti OR 'systematic review':de,ab,ti OR 'registry':de,ab,ti OR 'birth register':de,ab,ti OR 'observational study':de,ab,ti OR 'population-based health datasets':de,ab,ti OR 'population health data':de,ab,ti OR 'Observational Study':de,ab,ti OR 'Longitudinal Studies':de,ab,ti OR 'Registries':de,ab,ti OR 'Retrospective Studies':de,ab,ti OR 'retrospective study':de,ab,ti OR 'Randomized Controlled Trial':de,ab,ti OR 'matched controls':de,ab,ti OR 'matched control':de,ab,ti OR 'case and control':de,ab,ti OR 'compared with controls':de,ab,ti OR 'case-control':de,ab,ti OR 'healthy controls':de,ab,ti OR disproportionality:de,ab,ti OR 'proportional reporting ratio':de,ab,ti OR 'propensity score':de,ab,ti OR 'case control study':de,ab,ti OR 'controlled study':de,ab,ti) AND ('human'/exp OR human OR m?n OR wom?n OR child OR boy OR girl)
