# Supplementary material for: Risk of drug use during pregnancy: master protocol for living systematic reviews and meta-analyses performed in the metaPreg project
Source: Syst Rev. 2023 Jun 21;12:101. doi: 10.1186/s13643-023-02256-8 (PMC10286473; doi:10.1186/s13643-023-02256-8)
Supplement: Supplementary file 4 — Additional file 4. Pre-established list of potential confounders for each class of endpoints. [file 13643_2023_2256_MOESM4_ESM.pdf]

Additional file 4: Pre-established list of potential confounders for each class of endpoints (version 1)

Class of endpoint	Major potential confounders	Sources
Pregnancy loss (spontaneous abortions, stillbirths…)	- History of pregnancy loss<br>- SGA/IUGR/LBW<br>- Assisted reproductive technology<br>- Hypertensive disorders<br>- Diabete<br>- Maternal age<br>- Lupus erythematosus<br>- Placenta abruptio<br>- Chromosomal and Genetic Abnormalities<br>- Multiple gestation<br>- BMI<br>- Oligohydramnios<br>- Renal disease	The American College of Obstetricians and Gynecologists (ACOG) 2018<br><br>The American College of Obstetricians and Gynecologists (ACOG) 2020<br><br>Flenady 2011
Congenital malformations	- History of congenital malformations<br>- Diabete<br>- BMI<br>- Maternal age<br>- Co-exposure to teratogens (alcohol, drugs)<br>- Multiple gestation<br>- Maternal pathology	Harris 2017
Anthropometric parameters (SGA/IUGR/LBW…) and preterm	- Multiple pregnancy<br>- History of the outcome<br>- Maternal age<br>- Hypertensive disorders (chronic hypertension, pre-eclampsia and pregnancy-related hypertension)<br>- Smoking<br>- Parity<br>- Alcohol<br>- Use of illegal drugs<br>- Socio-economic status<br>- BMI<br>- Assisted reproductive technology<br>- History of elective termination of pregnancy	Sentilhes 2017<br>Di Renzo 2017<br>The American College of Obstetricians and Gynecologists (ACOG) 2019<br>The American College of Obstetricians and Gynecologists (ACOG) 2021<br>Vayssière 2015<br>Lausman 2013
Neuro-developmental disorders (ASD, ADHD, cognitive disorders, …)	- Gender/sex<br>- Ethnicity<br>- Socioeconomic status / Maternal IQ	Cogley 2021<br>Cénat 2021<br>Cheng 2019

Class of endpoint	Major potential confounders	Sources
	- Genetic and familial factor<br>- Brain anomaly<br>- Maternal age<br>- Hypertensive disorders (chronic hypertension, pre-eclampsia and pregnancy-related hypertension)<br>- BMI (maternal overweight before or during pregnancy)<br>- SGA, preterm (gestational age)<br>- Paternal age	Kim 2019
Maternal consequences (pre-eclampsia, gestational diabetes, …)	- History of the outcome<br>- Family history of the outcome<br>- Chronic pathology (hypertension, diabete, auto-immune disease…)<br>- Maternal age<br>- Parity<br>- BMI<br>- Multiple gestation	Teh 2011<br>Bartsch 2016<br>Giannakou 2019<br>NICE 2019

ASD: autism spectrum disorder; ADHD: attention deficit with or without hyperactivity disorder; BMI: body mass index; IQ: intellectual quotient; IUGR: intrauterine growth restriction; LBW: low birth weight; NICE: The National Institute for Health and Care Excellence; SGA: small for gestational age.

American College of Obstetricians and Gynecologists 2018 - Early pregnancy loss. ACOG Practice Bulletin No. 200. American College of Obstetricians and Gynecologists. Obstetrics & Gynecology;132:e197–207.

American College of Obstetricians and Gynecologists 2019 - Fetal growth restriction. ACOG Practice Bulletin No. 204. American College of Obstetricians and Gynecologists. Obstetrics & Gynecology; 133:e97–109.

American College of Obstetricians and Gynecologists 2020 - Management of stillbirth. ACOG Obstetric Care Consensus No. 10. American College of Obstetricians and Gynecologists. Obstetrics & Gynecology; 135:e110–132.

American College of Obstetricians and Gynecologists 2021 - Prediction and Prevention of Spontaneous Preterm Birth: ACOG Practice Bulletin No. 234. American College of Obstetricians and Gynecologists. Obstetrics & Gynecology; 138:e65–90.

Bartsch 2016 - Clinical risk factors for pre-eclampsia determined in early pregnancy: systematic review and meta-analysis of large cohort studies. British Medical Journal; 353:i1753.

Cénat 2021 - Prevalence and Risk Factors Associated With Attention-Deficit/ Hyperactivity Disorder Among US Black Individuals. A Systematic Review and Meta-analysis.Journal of American Medical Association Psychiatry; 78:21-28.

Cheng 2009 - Improving autism perinatal risk factors: A systematic review. Medical Hypotheses; 127:26–33.

Cogley 2021 - A Systematic Review of the Risk Factors for Autism Spectrum Disorder in Children Born Preterm. Child Psychiatry & Human Development; 52:841-855.

Di Renzo 2017 - Preterm Labor and Birth Management: Recommendations from the European Association of Perinatal Medicine. The Journal of Maternal-Fetal & Neonatal Medicine; 30:2011-2030.

Flenady 2011 - Major risk factors for stillbirth in high-income countries: a systematic review and meta-analysis. Lancet; 377:1331–1340.

Giannakou 2019 - Risk factors for gestational diabetes: An umbrella review of meta-analyses of observational studies. PLoS One;14:e0215372.

Harris 2017 - Risk factors for birth defect. Obstetrical and Gynecological Survey; 72:123-135.

Kim 2019 - Environmental risk factors and biomarkers for autism spectrum disorder: an umbrella review of the evidence. Lancet Psychiatry; 6:590–600.

Lausman 2013 - Intrauterine Growth Restriction: Screening, Diagnosis, and Management. Journal of Obstetrics and Gynaecology Canada; 35:741–748.

NICE 2019 - Hypertension in pregnancy: diagnosis and management. Available on: www.nice.org.uk/guidance/ng133.

Sentilhes 2017 - Prevention of spontaneous preterm birth: Guidelines for clinical practice from the French College of Gynaecologists and Obstetricians (CNGOF). European Journal of Obstetrics & Gynecology and Reproductive Biology; 210:217–224.

Teh 2011. Risk factors for gestational diabetes mellitus: Implications for the application of screening guidelines. Australian and New Zealand Journal of Obstetrics and Gynaecology; 51:26-30.

Vayssière 2015 - Fetal growth restriction and intra-uterine growth restriction: guidelines for clinical practice from the French College of Gynaecologists and Obstetricians. European Journal of Obstetrics & Gynecology and Reproductive Biology; 193:10–18.
