# Supplementary material for: Risk of drug use during pregnancy: master protocol for living systematic reviews and meta-analyses performed in the metaPreg project
Source: Syst Rev. 2023 Jun 21;12:101. doi: 10.1186/s13643-023-02256-8 (PMC10286473; doi:10.1186/s13643-023-02256-8)
Supplement: Supplementary file 5 — Additional file 5. Relevancy (Yes/No) of the period of exposures for each outcome. If the period of exposure is relevant (Yes), the results available in the publication for the considered outcome will be included in the meta-analysis, otherwise (No), the results available in the publication for the considered outcome will be excluded. [file 13643_2023_2256_MOESM5_ESM.pdf]

Additional file 5: Relevancy (Yes/No) of the period of exposures for each outcome. If the period of exposure is relevant (Yes), the results available in the publication for the considered outcome will be included in the meta-analysis, otherwise (No), the results available in the publication for the considered outcome will be excluded.

	During pregnancy (anytime or not specified)	1st trimester	2nd trimester	3rd trimester	2nd and/or 3rd trimester	Throughout pregnancy	Early pregnancy	Days before delivery	Late pregnancy	At least 1st trimester	1st and 2nd trimester	Preconception-only
**Congenital malformations**												
As a whole, subgroup or individual malformations (except the 3 below)	No	Yes	No	No	No	Yes	Yes	No	No	Yes	Yes	No
Craniosynostosis	Yes	Yes	Yes	Yes	Yes	Yes	Yes	No	Yes	Yes	Yes	No
Microcephaly	Yes	Yes	Yes	Yes	Yes	Yes	Yes	No	Yes	Yes	Yes	No
Hydrocephalus	Yes	Yes	Yes	Yes	Yes	Yes	Yes	No	Yes	Yes	Yes	No
**Growth parameters and prematurity**												
Preterm / Very preterm / Extremely preterm	Yes	Yes	Yes	Yes	Yes	Yes	Yes	Yes	Yes	Yes	Yes	No
Large for gestational age (weight)	Yes	Yes	Yes	Yes	Yes	Yes	Yes	No	Yes	Yes	Yes	No
Large for gestational age (length)	Yes	Yes	Yes	Yes	Yes	Yes	Yes	No	Yes	Yes	Yes	No
Large head circumference for gestational age	Yes	Yes	Yes	Yes	Yes	Yes	Yes	No	Yes	Yes	Yes	No
Small for gestational age (weight)	Yes	Yes	Yes	Yes	Yes	Yes	Yes	No	Yes	Yes	Yes	No
Small for gestational age (lenght)	Yes	Yes	Yes	Yes	Yes	Yes	Yes	No	Yes	Yes	Yes	No
Small head circumference for gestational age	Yes	Yes	Yes	Yes	Yes	Yes	Yes	No	Yes	Yes	Yes	No
Low birth weight (< 2500 g) / Very low birth weight (< 1500 g)	Yes	Yes	Yes	Yes	Yes	Yes	Yes	No	Yes	Yes	Yes	No
Macrosomia (> 4000 g)	Yes	Yes	Yes	Yes	Yes	Yes	Yes	No	Yes	Yes	Yes	No
**Intrauterine deaths**												
Early intrauterine death (< 22 weeks)	Yes	Yes	Yes	No	Yes	Yes	Yes	Yes	No	Yes	Yes	No
Ectopic pregnancy	No	Yes	No	No	No	Yes	Yes	No	No	Yes	No	No
Elective termination of pregnancy	Yes	Yes	Yes	Yes	Yes	Yes	Yes	Yes	Yes	Yes	Yes	No
Intrauterine deaths (as a whole)	Yes	Yes	Yes	Yes	Yes	Yes	Yes	Yes	Yes	Yes	Yes	No
Late intrauterine deaths (> 22 weeks)	Yes	Yes	Yes	Yes	Yes	Yes	Yes	Yes	Yes	Yes	Yes	No
Perinatal death	Yes	Yes	Yes	Yes	Yes	Yes	Yes	Yes	Yes	Yes	Yes	No
Therapeutic terminations of pregnancy	Yes	Yes	Yes	Yes	Yes	Yes	Yes	No	Yes	Yes	Yes	No
**Maternal consequences**												
Abruptio placentae	Yes	Yes	Yes	Yes	Yes	Yes	Yes	Yes	Yes	Yes	Yes	No
Assisted deliveries (forceps, vacuum, ...)	Yes	No	No	Yes	Yes	Yes	No	Yes	Yes	No	No	No
Caesarean	Yes	No	No	Yes	Yes	Yes	No	Yes	Yes	No	No	No
Gestational diabete	Yes	Yes	Yes	Yes	Yes	Yes	Yes	No	Yes	Yes	Yes	No
Maternal consequences (as a whole)	Yes	Yes	Yes	Yes	Yes	Yes	Yes	Yes	Yes	Yes	Yes	No
Maternal death	Yes	Yes	Yes	Yes	Yes	Yes	Yes	Yes	Yes	Yes	Yes	No
Maternal hypothyroidism	Yes	Yes	Yes	Yes	Yes	Yes	Yes	Yes	Yes	Yes	Yes	No
Maternal infections	Yes	Yes	Yes	Yes	Yes	Yes	Yes	No	Yes	Yes	Yes	No
Maternal liver disorder / failure during pregnancy	Yes	Yes	Yes	Yes	Yes	Yes	Yes	Yes	Yes	Yes	Yes	No
Oligohydramnios / Polyhydramnios	Yes	Yes	Yes	Yes	Yes	Yes	Yes	Yes	Yes	Yes	Yes	No
Placenta previa	No	Yes	No	No	No	Yes	Yes	No	No	Yes	Yes	No
Hydramnios	Yes	Yes	Yes	Yes	Yes	Yes	Yes	Yes	Yes	Yes	Yes	No
Postpartum hemorrhage	Yes	No	No	Yes	Yes	Yes	No	Yes	Yes	No	No	No
Preeclampsia	Yes	Yes	Yes	Yes	Yes	Yes	Yes	No	No	Yes	Yes	No
**Neonatal disorders**												
Bone fractures / bone mineralisation anomalies	Yes	Yes	Yes	Yes	Yes	Yes	Yes	No	Yes	Yes	Yes	No
Eye deficiency / Visual abnormality	Yes	Yes	Yes	Yes	Yes	Yes	Yes	No	Yes	Yes	Yes	No
Fetal distress	Yes	No	Yes	Yes	Yes	Yes	No	Yes	Yes	No	Yes	No
Goiter	Yes	No	Yes	Yes	Yes	Yes	No	Yes	Yes	No	Yes	No
Hearing loss / Auditory deficit	Yes	Yes	Yes	Yes	Yes	Yes	Yes	Yes	Yes	Yes	Yes	No
Hyperthyroidism / Thyrotoxicosis (fetal/neonatal)	Yes	No	Yes	Yes	Yes	Yes	No	No	Yes	No	Yes	No
Hypothyroidism (fetal/neonatal)	Yes	No	Yes	Yes	Yes	Yes	No	Yes	Yes	No	Yes	No
Child/Infant death (> 28 days of life)	Yes	Yes	Yes	Yes	Yes	Yes	Yes	Yes	Yes	Yes	Yes	No
Feeding difficulty	Yes	Yes	Yes	Yes	Yes	Yes	Yes	Yes	Yes	Yes	Yes	No
Infant growth abnormalities	Yes	Yes	Yes	Yes	Yes	Yes	Yes	No	Yes	Yes	Yes	No
Infant health status	Yes	Yes	Yes	Yes	Yes	Yes	Yes	Yes	Yes	Yes	Yes	No
Jaundice	Yes	Yes	Yes	Yes	Yes	Yes	Yes	Yes	Yes	Yes	Yes	No
Low Apgar score (< 7) (at 1 min ; at 5 min ; at 10 min)	Yes	No	No	Yes	Yes	Yes	No	Yes	Yes	No	No	No
Very low Apgar score (< 7) (at 1 min ; at 5 min ; at 10 min)	Yes	No	No	Yes	Yes	Yes	No	Yes	Yes	No	No	No
Metabolic disorders in neonate/infant	Yes	Yes	Yes	Yes	Yes	Yes	Yes	Yes	Yes	Yes	Yes	No
Mitochondrial abnormalities	Yes	Yes	Yes	Yes	Yes	Yes	Yes	No	Yes	Yes	Yes	No
Necrotizing enterocolitis	Yes	Yes	Yes	Yes	Yes	Yes	Yes	Yes	Yes	Yes	Yes	No
Neonatal antibiotic resistance	Yes	Yes	Yes	Yes	Yes	Yes	Yes	No	Yes	Yes	Yes	No
Neonatal death (< 28 days of life)	Yes	Yes	Yes	Yes	Yes	Yes	Yes	Yes	Yes	Yes	Yes	No
Neonatal disorders (as a whole)	Yes	Yes	Yes	Yes	Yes	Yes	Yes	Yes	Yes	Yes	Yes	No
Neonatal hyperthyrotropinemia	Yes	No	Yes	Yes	Yes	Yes	No	Yes	Yes	No	Yes	No
Neonatal hypertonia	Yes	Yes	Yes	Yes	Yes	Yes	Yes	Yes	Yes	Yes	Yes	No
Neonatal hypotonia	Yes	Yes	Yes	Yes	Yes	Yes	Yes	Yes	Yes	Yes	Yes	No
Neonatal hypotension	Yes	No	Yes	Yes	Yes	Yes	No	Yes	Yes	No	Yes	No
Neonatal immune dysfunction	Yes	Yes	Yes	Yes	Yes	Yes	Yes	Yes	Yes	Yes	Yes	No
Neonatal infections	Yes	Yes	Yes	Yes	Yes	Yes	Yes	Yes	Yes	Yes	Yes	No
Neonatal intracranial hemorrhage	Yes	Yes	Yes	Yes	Yes	Yes	Yes	Yes	Yes	Yes	Yes	No
Neonatal medical care	Yes	No	Yes	Yes	Yes	Yes	No	Yes	Yes	No	Yes	No
Neonatal tachypnea	Yes	No	Yes	Yes	Yes	Yes	No	Yes	Yes	No	Yes	No
Persistent pulmonary hypertension	Yes	No	Yes	Yes	Yes	Yes	No	No	Yes	No	Yes	No
Withdrawal syndrome / Neonatal abstinence syndrome	Yes	No	No	Yes	Yes	Yes	No	Yes	Yes	No	No	No
**Long term disorders (cancer, asthma, allergies …)**	Yes	Yes	Yes	Yes	Yes	Yes	Yes	Yes	Yes	Yes	Yes	No
**Neurodeveloppmental disorders**	Yes	Yes	Yes	Yes	Yes	Yes	Yes	Yes	Yes	Yes	Yes	No
